# Supplementary material for: Identification of Freezing-Responsive microRNAs and Their Targets in Chinese Jujube by Small RNA and Degradome Sequencing
Source: Int J Mol Sci. 2026 Sep 4;27(17):7912. doi: 10.3390/ijms27177912 (PMC13566890; doi:10.3390/ijms27177912)
Supplement: Supplementary file 1 [file ijms-27-07912-s001.zip › Supplementary Material S2/novel_miR_219.pdf]

Provisional ID	:	novel_miR_219
Score total	:	7168.2
Score for star read(s)	:	3.9
Score for read counts	:	7159.1
Score for mfe	:	3.6
Score for randfold	:	1.6
Score for cons. seed	:	
Total read count	:	14054
Mature read count	:	9107
Loop read count	:	31
Star read count	:	4916

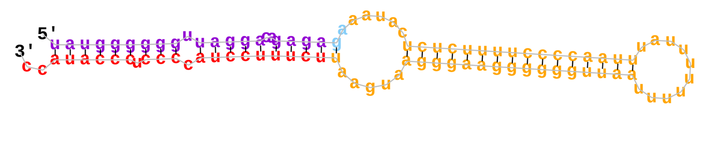

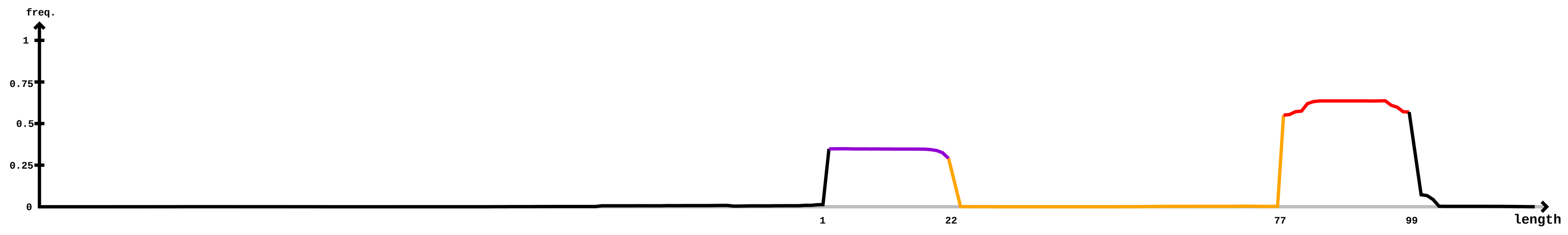

Star Mature

```
5'- uuccuuuuuuucaccaaaaucuagacucaauuaauuucucuuuucccuuccuccaccaucugguguauuucuuuucucacuaaaaggauggaagagggaaaggcuuaagagugugggugguggaagugauagguauggggguuaggacagagagaaauacucucuuuccccaauuuauuuuuuuuaauugggggaagggaaugaauucuuuccuaccccucccauaccauucauuuccauccuuuccuuc -3' obs
    uuccuuuuuuucaccaaaaucuagacucaauuaauuucucuuuucccuuccuccaccaucugguguauuucuuuucucacuaaaaggauggaagagggaaaggcuuaagagugugggugguggaagugauagguauggggguuaggacagagagaaauacucucuuuccccaauuuauuuuuuuuaauugggggaagggaaugaauucuuuccuaccccucccauaccauucauuuccauccuuuccuuc     exp
    ...................................((((((((((..((((((...(((((...........)))))...)).)))).))))))))))...(((.(...(((((((((((..((((((((((.(((((..(((((......(((((((((((((((.........))))))))))))))))).....))))))))))).))).)))))))))..))))))))))))...)))).
```

read	reads	mm	sample
caccaaaaucuagacucaauu	1	0	LT-1
ccaaaaucuagacucaauuaauuucucu	1	0	JCK-2
aaucuagacucaauuaauuucuc	1	0	LT-3
uagacucaauuaauuucucuuuuc	1	0	JCK-1
agacucaauuaauuucucuuuucc	1	0	JCK-2
agacucaauuaauuucucuuuucc	1	0	JCK-1
agacucaauuaauuucucuuuucc	1	0	JCK-3
ucugguguauuucuuuucuc	2	0	JCK-3
ucucacuaaaaggauggaagagggaa	2	0	LT-1
ucucacuaaaaggauggaagagggaaagg	1	0	JCK-3
ucucacuaaaaggauggaagagggaaagg	1	0	JCK-1
cucacuaaaaggauggaagagggaaagg	1	0	JCK-3
cacuaaaaggauggaagagg	1	0	LT-3
cacuaaaaggauggaagagggaaagg	1	0	JCK-2
cacuaaaaggauggaagagggaaaggA	2	1	JCK-3
cacuaaaaggauggaagagggaaaggA	2	1	JCK-2
aaaaggauggaagagggaaagg	4	0	LCK-3
aaaaggauggaagagggaaagg	1	0	LT-3
aaaggauggaagagggaaagg	1	0	LT-3
aggauggaagagggaaagg	3	0	LT-2
agagggaaaggcuuaagagu	5	0	LT-2
agagggaaaggcuuaagagug	1	0	LT-3
agagggaaaggcuuaagagugu	36	0	LCK-2
agagggaaaggcuuaagagugu	21	0	LCK-1
gagggaaaggcuuaagagug	1	0	JCK-2
Cgggaaaggcuuaagaguguggg	1	1	LT-1
aaaggcuuaagagugugggugg	1	0	LT-3
aaaggcuuaagagugugggguggaagu	2	0	JCK-1
aggcuuaagagugugggug	1	0	LT-1
cuuaagagugugggguggaaguga	2	0	JT-2
cuuaagagugugggguggaaguga	2	0	JT-3
cuuaagagugugggguggaagugauag	2	0	JCK-2
cuuaagagugugggguggaagugauag	2	0	JCK-3

Star (purple) **Mature** (red)

uuccuuuuuuucaccaaaaucuagacucaauuaauuucucuuuucccuuccuccaccaucugguguauuucuuuucucacuaaaaggauggaagagggaaaggcuuaagagugugggguggaagugauagguaugggggguuaggacagagagaaauacucucuuuuccccaauuuauuuuuuuuaauuggggggaagggaaugaauucuuuccuaccccucccauaccauucauuuccauccuuuccuuc

Read	Count	Mismatch	Sample
cuuaagagugugggguggaagugauagg	1	0	JCK-3
cuuaagagugugggguggaagugauagg	1	0	LT-2
cuuaagagugugggguggaagugauagg	2	0	LT-1
cuuaagagugugggguggaagugauagg	4	0	JT-2
cuuaagagugugggguggaagugauagg	3	0	JT-3
cuuaagagugugggguggaagugauagg	5	0	JCK-2
aagagugugggguggaagugauagg	4	0	JT-3
aagagugugggguggaagugauagg	2	0	JT-2
gugugggguggaagugauagg	1	0	JCK-3
gugugggguggaagugauagg	1	0	JCK-1
ugugggguggaagugauaggu	10	0	LCK-3
gugggguggaagugauagguau	1	0	LT-1
gugggguggaagugauagguaug	8	0	LCK-3
uggggguggaagugauagguaugg	2	0	JCK-3
gguggaagugauagguauggg	1	0	LT-3
guggaagugauagguaug	5	0	LCK-3
guggaagugauagguauggg	1	0	LT-1
guggaagugauagguaugggg	2	0	JCK-2
guggaagugauagguaugggg	1	0	JCK-3
uggaagugauagguaugg	1	0	LT-3
uggaagugauagguaugggg	2	0	JCK-2
uggaagugauagguaugggg	1	0	JCK-3
uggaagugauagguauggggggg	6	0	LCK-3
agugauagguaugggggguu	3	0	LT-3
agugauagguaugggggguuaggaca	7	0	LCK-3
agugauagguaugggggguuaggacagaga	2	0	LCK-3
ugauagguaugggggguuaggaca	3	0	LT-3
auagguaugggggguuaggacaga	1	0	JCK-1
auagguaugggggguuaggacaga	1	0	JCK-2
uagguaugggggguuagga	4	0	LT-3
uagguaugggggguuagga	1	0	JCK-1
uagguaugggggguuaggac	2	0	JCK-2
uagguaugggggguuaggacC	1	1	LT-3
uagguaugggggguuaggaca	4	0	LCK-3
uagguaugggggguuaggaca	1	0	LT-3
uagguaugggggguuaggaca	6	0	JCK-3
uagguaugggggguuaggacC	2	1	JCK-3
uagguaugggggguuaggaca	6	0	JCK-2
uagguauggggAuuaggaca	1	1	LT-1
uagguaugggggguuaggacU	1	1	LT-3
uagguaugggggguuaggaca	1	0	JCK-1
uagguaugggggguuaggaca	3	0	JT-3
uagguaugggggguuaggaca	1	0	JT-2
uagguaugggggguuaggaca	3	0	LT-1
uagguaugggggguuaggacaga	1	0	JCK-3
gguaugggggguuaggacag	1	0	JCK-3
gguaugggggguuaggacaga	4	0	LT-1
gguaugggggguuaggacaga	1	0	LCK-3
gguaugggggguuaggacaga	3	0	JCK-2
gguaugggggguuaggacaga	9	0	JCK-1
gguaugggggguuaggacaga	4	0	JCK-3
gguaugggggguuaggacaga	9	0	LT-2
gguaugggggguuaggacaga	3	0	LT-3
gguaugggggguuaggacaga	12	0	JT-1
guaugggggguuaggacaga	1	0	LT-3
guaugggggguuaggacaga	2	0	LT-1
guaugggggguuaggacaga	6	0	LCK-3
guaugggggguuaggacagag	1	0	JCK-3
Cuaugggggguuaggacagaga	2	1	JCK-2
Cuaugggggguuaggacagaga	1	1	LT-3
Cuaugggggguuaggacagaga	2	1	JCK-1
Cuaugggggguuaggacagaga	7	1	JT-1
uaugggggguuaggacag	14	0	JCK-2
uaugggggguuaggacag	10	0	LCK-3
uaugggggguuCggacag	1	1	JCK-2
uaugggggguuaggacag	10	0	LT-3
uaugggggguuaggacag	18	0	JCK-3
uaugggggguuaggacag	8	0	LT-1
uaugggggguuaggacag	8	0	LT-2
uaugggggguuaggacag	9	0	JCK-1

Star (uaugggggguuaggacagaga) — Mature (ucuuuccuaccccucccauacc)

uuccuuuuuuucaccaaaaucuagacucaauuaauuucucuuuucccuuccuccaccaucugguguauuucuuuucucacuaaaaggauggaagagggaaaggcuuaagagugugggguggaagugauagguaugggggguuaggacagagagaaauacucucuuuuccccaauuuauuuuuuuuaauuggggggaagggaaugaauucuuuccuaccccucccauaccauucauuuccauccuuuccuuc

uaugggggguuaggacaga	12	0	JCK-3
uaugggggguuaggacaga	11	0	JT-1
uNugggggguuaggacaga	1	1	JT-2
uaugggggguuaggacaga	8	0	LT-1
uaugggggguuaggacagU	1	1	LT-1
uaugggggguuaggacaga	14	0	LT-3
uaugggggguuaggacagU	1	1	JCK-2
uaugggggguuaggacaga	19	0	LT-2
uaugggggguuaggacaga	37	0	LCK-3
uaugggggguuaggacaga	11	0	JCK-2
uaugggggguuaggacaga	5	0	JT-3
uCugggggguuaggacaga	1	1	JCK-1
uaugggggguuaggacaga	11	0	JT-2
uaugggggguuaggacagag	43	0	LT-3
uaugggggguuaggacagaN	1	1	JCK-1
uaugggggguuaggacagag	20	0	JT-3
uaugggggguuaggacagag	35	0	LT-2
uaugggggguuaggacagag	115	0	JT-1
uaugggggguuaggacagag	7	0	JT-2
uCugggggguuaggacagag	3	1	JCK-2
uaugggggguuaggacagag	60	0	JCK-1
uCugggggguuaggacagag	1	1	JT-3
uaugggggguuaggacagag	62	0	JCK-3
uaugggggguuCggacagag	1	1	JCK-2
uaugggggguuaggacagag	34	0	LT-1
uaugggggguuaggacagaU	1	1	LT-2
uaugggggguuNggacagag	1	1	JCK-1
uaugggggguuaggacagag	16	0	LCK-3
uaugggggguuaggacagag	91	0	JCK-2
uaugggggguuaggacagaNa	1	1	JCK-1
uaugggggguuaggacagaga	345	0	LCK-3
uaugggggguuaggacagaga	113	0	LCK-1
uaugggggguuaggacagCga	1	1	LT-2
uaugggggguuaggacagaga	589	0	JT-1
uaugggggguuaggacagaga	242	0	LT-2
uauAggggguuaggacagaga	1	1	JCK-1
uCugggggguuaggacagaga	1	1	JCK-3
uaugggggguuaggCcagaga	1	1	LCK-1
uaugggggguuagAacagaga	1	1	LCK-3
uaugggggguuCggacagaga	1	1	JCK-3
uaugggggguuCggacagaga	1	1	LCK-2
Caugggggguuaggacagaga	1	1	LT-2
uaugggggguuaggacagagC	2	1	JCK-1
uaugggggguuagUacagaga	1	1	LCK-2
uaugggggguuaggacagaga	478	0	JCK-1
uaugggggguuaggacagagU	1	1	JCK-1
uaugggggguuaggacagaga	555	0	JCK-3
uCugggggguuaggacagaga	4	1	LT-2
uNugggggguuaggacagaga	1	1	JCK-2
uaugggggguuaggacagaga	727	0	JCK-2
uCugggggguuaggacagaga	3	1	JT-1
uaugggggguuaggacagaga	192	0	JT-2
uaugggggguuaggCcagaga	1	1	JCK-1
uauAggggguuaggacagaga	1	1	JT-1
uaCggggguuaggacagaga	1	1	JT-3
uCugggggguuaggacagaga	2	1	LCK-3
uaugggggguuaggCcagaga	2	1	JCK-2
uaugggggguuNggacagaga	1	1	JCK-1
uaNggggguuaggacagaga	1	1	LCK-2
uaugggggguuaggacagaga	289	0	LT-3
uauAggggguuaggacagaga	1	1	JCK-2
uaugggggguuaggacagagC	2	1	JCK-3
uauAggggguuaggacagaga	1	1	JCK-3
uaugggggguuaggCcagaga	1	1	LT-3
uaugggggguuaggCcagaga	1	1	JT-1
uCugggggguuaggacagaga	1	1	JT-3
uaugggggguuagNacagaga	2	1	LCK-1
uaugggggguuaggacagaga	88	0	LCK-2
uaugggggguuaggacagagU	1	1	LT-1
uaugggggguuaggCcagaga	1	1	JT-2

Star　　　　　　　　　　　　　　　　　　Mature

Sequence			
uuccuuuuuuucaccaaaaucuagacucaauuaauuucucuuuucccuuccuccaccaucugguguauuucuuuucucacuaaaaggauggaagagggaaaggcuuaagagugugguggaagugauagg**uaugggggguuaggacagaga**gaaauacucucuuuuccccaauuuauuuuuuuuaauuggggggaagggaaugaau**ucuuuccuaccccucccauacc**auucauuuccauccuuuccuuc			
uaugggggguuaggacagaga	231	0	JT-3
uaugggggguuaggacagaga	206	0	LT-1
uaugggggguuaggacCgaga	1	1	JCK-2
uaugggggguuaggCcagaga	1	1	JCK-3
uCugggggguuaggacagaga	6	1	JCK-2
uaugggggGCuaggacagaga	1	1	JT-2
uaugggggguuCggacagaga	1	1	LCK-3
uaugggUgguuaggacagaga	1	1	JT-3
uaugggggguuaggacagagU	3	1	LT-3
uaugggggguuaggacagagaU	1	1	LT-2
uaugggggguuaggacagagaU	1	1	JCK-2
uaugggggguuaggacagagaU	1	1	LT-3
uaugggggguuaggacagagaC	1	1	JCK-3
uaugggggguuaggacagagaC	3	1	JCK-2
uaugggggguuaggacagagaU	2	1	LT-1
uaugggggguuaggacagagagaa	2	0	JCK-2
augggggguuaggacaga	2	0	LT-2
augggggguuaggacaga	1	0	LT-3
augggggguuaggacagag	3	0	JCK-1
augggggguuaggacagag	1	0	JCK-2
augggggguuaggacagag	1	0	LT-2
augggggguuaggacagaga	2	0	JCK-2
augggggguuaggacagaga	5	0	JCK-3
augggggguuaggacagagaU	1	1	JCK-2
ugggggguuaggacagaga	3	0	JCK-2
ugggggguuaggacagaga	1	0	JCK-1
gggggguuaggacagagagaaaua	1	0	LT-3
ggggguuaggacagagagaaauac	1	0	LT-3
gggguuaggacagagagaaauac	4	0	LCK-3
guuaggacagagagaaauacu	3	0	LCK-3
acucucuuuuccccaauuuUuuuuuu	1	1	LT-1
ucucuuuuccccaauuuauA	1	1	LT-3
uuuuuaauuggggggaaggga	5	0	LCK-3
uuuuuaCuuggggggaaggga	1	1	LCK-3
uuaauuggggggaagggaau	1	0	LT-3
uuaauuggggggaCgggaaug	1	1	LT-1
uuaauuggggggaagggaaug	1	0	LT-1
uaauuggggggaagggaaugaau	1	0	LT-3
uaauuggggggaagggaaugaauA	6	1	LCK-3
uaauuggggggaagggaaugaauuc	2	0	LT-3
uaauuggggggaagggaaugaauuc	2	0	LCK-3
aauuggggggaagggaau	6	0	LCK-3
uuggggggaagggaaugaauucuuuc	7	0	LCK-3
ugggggggaagggaaugaauucu	1	0	JCK-2
ugggggggaagggaaugaauucu	1	0	JCK-1
ggggaagggaaugaauucu	1	0	LT-1
aaugaauucuuuccuaccccuc	4	0	JT-2
aaugaauucuuuccuaccccuc	7	0	JT-3
Cucuuuccuaccccuccca	1	1	LT-1
ucuuuccuaccccuccca	3	0	JT-1
ucuuuccuaccccuccca	40	0	LT-1
ucuuuccuaccccuccca	35	0	LCK-2
ucuuuccuaccccuccca	67	0	LCK-3
ucuuCccuaccccuccca	1	1	LT-2
ucuuuccuUccccuccca	3	1	LT-2
ucuuuccuaccccNccca	1	1	LCK-1
ucuuuccuaccccuccca	88	0	LT-2
ucuuuccuaccccuccca	33	0	LCK-1
ucuuuccuaccccuccca	50	0	LT-3
ucuuuccuaccccuccca	9	0	JT-3
ucuuuccuaccccuccca	22	0	JCK-2
ucuuuccuacAccuccca	1	1	LCK-3
ucuuuccuaccccucccU	1	1	LCK-3
ucuuuccuaccccuccca	9	0	JT-2
ucuuuccuaccccuccca	9	0	JCK-3
ucuuuccuaccccuccca	16	0	JCK-1
ucuuuccuaccccuAccau	1	1	LCK-3
ucuuuccuaccccucccau	25	0	LT-3
ucuuuccuaccccuGccau	1	1	LCK-1
ucuuuccuaccccucccau	14	0	LT-2

	Star	Mature			
uuccuuuuuuucaccaaaaucuagacucaauuaauuucucuuuucccuuccuccaccaucugguguauuucuuuucucacuaaaaggauggaagagggaaaggcuuaagaguguggguggaagugauagg	uauggggguuaggacagaga (purple)<br>gaaauacucucuuuuccccaauuuauuuuuuuuaauugggggaagggaaugaau (orange)	ucuuuccuaccccucccauacc (red)<br>auucauuuccauccuuuccuuc			
		ucuuuccuaccccucccau	6	0	JCK-3
		ucuuuccuaccccucccau	17	0	LT-1
		ucuuuccuaccccucccau	26	0	LCK-2
		ucuuuccuaccccucccau	23	0	LCK-3
		ucuuuccuaccccucccau	27	0	LCK-1
		ucuuuccuaccccucccau	14	0	JCK-2
		ucuuuccuaccccucccau	12	0	JCK-1
		ucuuuccuaccccucccCua	1	1	LT-3
		ucuuuccuacUccucccaua	28	1	JT-1
		ucuuuccuaccccucccaua	35	0	LT-3
		uNuuuccuaccccucccaua	1	1	JCK-2
		ucuuuccuaccccucccauU	2	1	LT-3
		ucuuuccuaccccucccauU	2	1	LT-1
		ucuuuccuaccccucccauC	2	1	LT-3
		ucuuuccuaccccucccaua	41	0	LT-2
		ucuuuccuaccccucccaua	53	0	LCK-3
		ucuuuccuaccccucccaAa	1	1	JCK-3
		ucuuuccuaccccucccaua	43	0	JCK-1
		ucuuuccuaccccucccaua	42	0	LT-1
		ucuuuccuaccccucccaua	57	0	JCK-3
		ucuuuccuaccccucccaua	59	0	JCK-2
		ucuuuccuaccccucccauac	1	0	JCK-1
		ucuuuccuaccccucccauac	7	0	JCK-2
		ucuuuccuaccccucccauac	8	0	LT-2
		ucuuuccuaccccucccauac	5	0	JCK-3
		ucuuuccuaccccucccauac	4	0	LT-3
		ucuuuccuaccccucccauac	3	0	LT-1
		ucuuuccuaccccucccauaA	1	1	LT-1
		ucuuuccuaccccucccauCcc	3	1	JCK-2
		ucuuuccuaccccucccauaGc	1	1	JCK-1
		ucuuCccuaccccucccauacc	1	1	JCK-3
		Ccuuuccuaccccucccauacc	2	1	LT-2
		ucuuuccuaccccucccauacc	1146	0	JCK-1
		ucuuuccuacUccucccauacc	80	1	LCK-2
		ucuuuccuacUccucccauacc	14	1	JT-2
		ucuuuccuaccccucccauacc	29	0	JT-2
		uUuuuccuaccccucccauacc	1	1	JCK-3
		ucuuuccuaccccucccauCcc	2	1	LT-3
		ucuuuccuaccccucccauCcc	1	1	LT-1
		ucuuuccuaccccucccCuacc	2	1	JCK-3
		ucuuuccuaccccucccauacU	2	1	JCK-1
		ucCuuccuaccccucccauacc	1	1	JCK-2
		ucuuuccuCccccucccauacc	3	1	JCK-3
		ucCuuccuaccccucccauacc	1	1	LT-2
		ucuuuccuaccccuUccauacc	1	1	JCK-3
		ucuuuccuacUccucccauacc	1	1	JCK-2
		ucuCuccuaccccucccauacc	1	1	LT-3
		ucuuuccuacUccucccauacc	114	1	JT-1
		ucuuuccuaccccucccauacc	1673	0	JCK-3
		ucuuuccuacGccucccauacc	3	1	JCK-3
		ucuuuccuacGccucccauacc	2	1	JCK-2
		ucuuuccuaccccuccUauacc	1	1	JCK-2
		ucuuuccuaccccCcccauacc	1	1	JCK-2
		ucuuuccuacUccucccauacc	87	1	LCK-1
		uNuuuccuaccccucccauacc	1	1	JCK-1
		ucuuuccuaccccucccauacc	48	0	JT-1
		ucuuuUcuaccccucccauacc	4	1	JCK-2
		Ccuuuccuaccccucccauacc	3	1	JCK-2
		ucuuuccuaccccucccauCcc	1	1	JCK-1
		ucuuuccuacccAucccauacc	1	1	LCK-3
		ucuuuccuCccccucccauacc	1	1	LT-1
		ucuuuUcuaccccucccauacc	1	1	JCK-1
		ucuuuccuaccccuUccauacc	4	1	JCK-1
		ucuuuccuaccccucccGuacc	1	1	JCK-1
		ucuuuccuaccccucccauaUc	1	1	JCK-2
		ucCuuccuaccccucccauacc	1	1	JCK-1
		ucuuuccuaccccucccauacc	26	0	LCK-1
		ucuuuccuaccccucccauCcc	1	1	LT-2
		ucCuuccuaccccucccauacc	1	1	JCK-3
		ucuuuccuCccccucccauacc	7	1	JCK-2

Star **Mature**

```
uuccuuuuuuucaccaaaaucuagacucaauuaauuucucuuuucccuuccuccaccaucugguguauuucuuuucucacuaaaaggauggaagagggaaaggcuuaagagugugggugguggaagugauagguaugggggguuaggacagagagaaauacucucuuuuccccaauuuauuuuuuuuaauugggggaagggaaugaauucuuuccuaccccucccauaccauucauuuccauccuuuccuuc
..............................................................................................................................................................................................................ucuuuccuaccccucccauacc......................   2190   0   JCK-2
..............................................................................................................................................................................................................ucuuuccuaccccucccauacc......................   18     0   JT-3
..............................................................................................................................................................................................................ucuuuccuaccccucccauaUc......................   1      1   JCK-3
..............................................................................................................................................................................................................ucuuuccuaccccucccauacc......................   214    0   LCK-3
..............................................................................................................................................................................................................ucuuuccuaccccucccauaGc......................   1      1   JCK-2
..............................................................................................................................................................................................................ucuuuccuaccccucccauacA......................   1      1   JCK-2
..............................................................................................................................................................................................................ucuuuccuaccccucccCuacc......................   6      1   JCK-2
..............................................................................................................................................................................................................ucuuuccuaAcccucccauacc......................   2      1   JCK-1
..............................................................................................................................................................................................................ucuuuccuaccccuccUauacc......................   2      1   LT-3
..............................................................................................................................................................................................................ucuGuccuaccccucccauacc......................   1      1   JCK-1
..............................................................................................................................................................................................................uNuuuccuaccccucccauacc......................   1      1   JCK-3
..............................................................................................................................................................................................................ucuuuccuaccccucccauacc......................   419    0   LT-3
..............................................................................................................................................................................................................uNuuuccuaccccucccauacc......................   4      1   JCK-2
..............................................................................................................................................................................................................ucuuucUuaccccucccauacc......................   3      1   JCK-2
..............................................................................................................................................................................................................ucuuuccuaccccucccauacc......................   434    0   LT-1
..............................................................................................................................................................................................................ucuuuccuacUccucccauacc......................   26     1   JT-3
..............................................................................................................................................................................................................Ccuuuccuaccccucccauacc......................   1      1   JCK-3
..............................................................................................................................................................................................................ucuuuccuaccNcucccauacc......................   3      1   JCK-1
..............................................................................................................................................................................................................ucuuuccuaccccucccauNcc......................   1      1   JCK-1
..............................................................................................................................................................................................................ucuuuccuacGccucccauacc......................   2      1   JCK-1
..............................................................................................................................................................................................................ucuuuccuaccccucccCuacc......................   4      1   JCK-1
..............................................................................................................................................................................................................ucuuuccuaccccucccCuacc......................   1      1   LT-2
..............................................................................................................................................................................................................ucuuuccuaccccucccauacU......................   1      1   LT-1
..............................................................................................................................................................................................................ucuuucUuaccccucccauacc......................   1      1   JCK-1
..............................................................................................................................................................................................................ucuuuccuaccccucccauacc......................   18     0   LCK-2
..............................................................................................................................................................................................................ucuuuccuCcccucccauacc.......................   2      1   LT-3
..............................................................................................................................................................................................................ucuuuccuaccccucUcauacc......................   1      1   LCK-3
..............................................................................................................................................................................................................uUuuuccuaccccucccauacc......................   1      1   JCK-1
..............................................................................................................................................................................................................ucuuuccuaUcccucccauacc......................   3      1   LT-3
..............................................................................................................................................................................................................ucuuuccuaccccucccauacc......................   226    0   LT-2
..............................................................................................................................................................................................................uUuuuccuaccccucccauacc......................   3      1   JCK-2
..............................................................................................................................................................................................................ucuuuccuaccccucccauacca.....................   1      0   JCK-1
..............................................................................................................................................................................................................ucuuuccuaccccucccauaccU.....................   11     1   LT-1
..............................................................................................................................................................................................................ucuuuccuaccccucccauaccU.....................   2      1   JCK-2
..............................................................................................................................................................................................................ucuuuccuaccccucccauaccU.....................   5      1   LT-3
..............................................................................................................................................................................................................ucuuuccuaccccucccauacca.....................   1      0   JCK-2
..............................................................................................................................................................................................................ucuuuccuaccccucccauaccU.....................   2      1   JCK-3
..............................................................................................................................................................................................................ucuuuccuaccccucccauacca.....................   2      0   LT-3
..............................................................................................................................................................................................................ucuuuccuaccccucccauaccU.....................   6      1   JCK-1
..............................................................................................................................................................................................................ucuuuccuaccccucccauacca.....................   1      0   LT-1
..............................................................................................................................................................................................................ucuuuccuaccccucccauaccUu....................   2      1   LT-2
..............................................................................................................................................................................................................ucuuuccuaccccucccauaccaA....................   3      1   LT-2
..............................................................................................................................................................................................................ucuuuccuaccccucccauaccUu....................   6      1   LCK-3
..............................................................................................................................................................................................................ucuuuccuaccccucccauaccUuu...................   3      1   LT-2
..............................................................................................................................................................................................................ucuuuccuaccccucccauaccUuu...................   1      1   LT-3
..............................................................................................................................................................................................................ucuuuccuaccccucccauaccaCuc..................   1      1   JCK-2
..............................................................................................................................................................................................................ucuuuccuaccccucccauaccauuG..................   1      1   LT-1
...............................................................................................................................................................................................................cuuuccuaccccucccau.........................   2      0   LCK-3
...............................................................................................................................................................................................................cuuuccuaccccucccau.........................   1      0   JCK-3
...............................................................................................................................................................................................................cuuuccuaccccucccaua........................   1      0   JCK-3
...............................................................................................................................................................................................................cuuuccuaccccucccaua........................   1      0   JCK-1
...............................................................................................................................................................................................................cuuuccuaccccucccaua........................   4      0   LCK-3
...............................................................................................................................................................................................................cuuuccuaccccucccauacc......................   6      0   LT-3
...............................................................................................................................................................................................................cuuuccuaccccucccauacc......................   11     0   JCK-1
...............................................................................................................................................................................................................cuuuccuaccccucccauacc......................   16     0   JCK-3
...............................................................................................................................................................................................................cuuuccuaccccucccauacc......................   11     0   JCK-2
...............................................................................................................................................................................................................cuuuccuaccccucccauaccauuU..................   2      1   JCK-3
...............................................................................................................................................................................................................cuuuccuaccccucccauaccauuU..................   2      1   JCK-2
................................................................................................................................................................................................................uuuccuaccccucccaua........................   1      0   LT-1
................................................................................................................................................................................................................uuuccuaccccucccaua........................   4      0   LCK-3
................................................................................................................................................................................................................uuuccuaccccucccaua........................   2      0   LT-3
................................................................................................................................................................................................................uuuccuaccccucccaua........................   1      0   JCK-2
................................................................................................................................................................................................................uuuccuaccccucccaua........................   1      0   LT-2
................................................................................................................................................................................................................uuuccuaccccucccaua........................   5      0   JCK-3
................................................................................................................................................................................................................uuuccuaccccucccauU........................   1      1   LT-3
................................................................................................................................................................................................................uuuccuaccccucccauacc......................   33     0   LT-1
................................................................................................................................................................................................................uuuccuCcccucccauacc.......................   2      1   LT-3
................................................................................................................................................................................................................uuuccuaccccucccauacU......................   1      1   LT-1
................................................................................................................................................................................................................uuuccuaccccucccauacc......................   12     0   JCK-1
................................................................................................................................................................................................................uuuccuaccccucccauacc......................   10     0   JCK-3
```

Star **Mature**

```
uuccuuuuuuucaccaaaaucuagacucaauuaauuucucuuuucccuuccuccaccaucugguguauuucuuuucucacuaaaaggauggaagagggaaaggcuuaagagugugggugggaagugauagguauggggguuaggacagagagaaauacucucuuuuccccaauuuauuuuuuuuaauugggggaagggaaugaauucuuuccuaccccucccauaccauucauuuccauccuuuccuuc
```

uuuccuacccucccauacc	28	0	LT-3
uuuccuacccucccauacc	8	0	LT-2
uuuccuacccucccauacc	18	0	JCK-2
Cuuccuacccucccauacc	2	1	LT-2
uuuccuacccucccauacc	11	0	LCK-3
uuuccuacccucccauacca	2	0	JCK-3
uuuccuacccucccauaccU	1	1	JCK-2
uuuccuacccucccauacca	1	0	JCK-1
uuuccuacccucccauacca	1	0	LT-3
uuuccuacccucccauacca	2	0	LT-1
uuuccuacccucccauacca	3	0	LCK-3
uuuccuacccucccauaccU	1	1	JCK-3
uuuccuacccucccauaccU	1	1	JCK-1
uuuccuacccucccauaccU	2	1	LT-1
uuuccuacccucccauacca	1	0	JCK-2
uuuccuacccucccauacca	2	0	JT-1
uNuccuacccucccauaccau	1	1	JCK-2
uuuccuacccAucccauaccau	1	1	JCK-2
uuuccuacccucccauaccau	5	0	JT-2
uuuccuacccucccauaccau	7	0	LT-3
uuuccuacccucccauaccau	4	0	LT-1
uuuccuacccucccauaccUu	1	1	LT-1
uuuccuacccucccauaccau	2	0	LCK-3
uuuccuacccucccauaccau	6	0	JT-3
uuuccuacccucccauaccau	10	0	JCK-1
uuuccuacccucccauaccau	15	0	JCK-2
uuuccuacccucccauaccau	7	0	JCK-3
uuuccuacccucccauaccauu	2	0	LT-2
uuuccuaccccNcccauaccauu	1	1	JCK-1
uuuccuacccucccauaccauu	3	0	LT-1
uuuccuacccucccauaccauu	3	0	JCK-2
uuuccuacccucccauaccauu	2	0	JCK-3
uuuccuacccucccauaccUuu	2	1	LT-2
uuuccuacccGucccauaccauu	1	1	LT-2
uuuccuacccucccauaccauu	3	0	LT-3
uuuccuacccucccauaccauuU	1	1	LT-1
uuuccuacccucccauaccauuc	1	0	LT-3
uuuccuacccucccauaccauuc	3	0	LT-1
uuuccuacccucccauaccauuc	1	0	LCK-3
uuuccuacccucccauaccauuc	3	0	JCK-2
uuuccuacccucccauaccauuc	1	0	JCK-1
uuuccuacccucccauaccauuc	6	0	JCK-3
uuccuacccucccauacc	1	0	LT-3
uuccuacccucccauacc	3	0	LT-1
uuccuacccucccauacc	6	0	LCK-3
uuccuacccucccauacc	8	0	JT-1
uuccuacccucccauaccauu	5	0	LCK-3
uuccuacccucccauaccauu	2	0	JCK-3
uAccuacccucccauaccauu	2	1	JCK-3
uAccuacccucccauaccauu	2	1	JCK-1
uuccuacccucccauaccauu	1	0	JCK-2
uuccuacccucccauaccauu	2	0	LT-1
uuccuacccucccauaccauuc	4	0	JCK-1
uuccuacccucccauaccauuc	4	0	JCK-3
uuccuacccucccauaccauuU	1	1	LT-1
uuccuacccucccauaccauuc	11	0	JCK-2
uuccuacccucccauaUcauuc	1	1	JCK-2
uccuacccucccauacc	5	0	JT-3
uccuacccucccauacc	1	0	LT-1
uccuacccucccauacc	1	0	LT-2
uccuacccucccauacc	1	0	JCK-2
uccuacccucccauacc	3	0	JT-2
uccuacccucccauacc	2	0	LT-3
uccuacccucccauacc	1	0	JCK-1
uccuacccucccauacc	2	0	LCK-3
uccuacccucccauacca	5	0	LT-2
uccuacccucccauacca	5	0	JT-3
uccuacccucccauaccauu	53	0	JCK-3
uccuacccucccauaccauu	20	0	LT-1
uccuacccucccauaccauu	52	0	JCK-1

```
                                                                                                                                  Star                                                                            Mature
uuccuuuuuuucaccaaaaucuagacucaauuaauuucucuuuucccuuccuccaccaucugguguauuucuuuucucacuaaaaggauggaagagggaaaggcuuaagagugugggguggaagugauagguaugggggguuaggacagagagaaauacucucuuuuccccaauuuauuuuuuuuaauugggggggaagggaaugaauucuuuccuaccccucccauaccauucauuuccauccuuuccuuc
...................................................................................................................................................................................................................uccuaccccucccauaccCuu...................   1    1    JCK-1
...................................................................................................................................................................................................................uccuaccccucccauaccauu...................   5    0    LT-2
...................................................................................................................................................................................................................uccuaccccucccauaccauu...................   11   0    LT-3
...................................................................................................................................................................................................................uccuacccAucccauaccauu...................   1    1    JCK-2
...................................................................................................................................................................................................................uNcuaccccucccauaccauu...................   1    1    JCK-3
...................................................................................................................................................................................................................uccuaccccucccaCaccauu...................   1    1    JCK-2
...................................................................................................................................................................................................................uccuaccccucccauaccauu...................   85   0    JCK-2
...................................................................................................................................................................................................................Cccuaccccucccauaccauuc..................   1    1    JCK-2
...................................................................................................................................................................................................................uccuaccccucccauaccauuc..................   142  0    JCK-2
...................................................................................................................................................................................................................uccuaccccucccauaccauuc..................   101  0    JCK-3
...................................................................................................................................................................................................................uccuaccccucccauaccauuc..................   8    0    LCK-3
...................................................................................................................................................................................................................uccuaccccucccauaccauuU..................   6    1    JCK-2
...................................................................................................................................................................................................................uccuaccccucccauaccauuc..................   5    0    LT-2
...................................................................................................................................................................................................................uccuaccccucccauaccauuU..................   1    1    LT-1
...................................................................................................................................................................................................................uccuaccccucccauaccauuc..................   16   0    LT-1
...................................................................................................................................................................................................................uccuaccccucccauaccauuU..................   3    1    JT-2
...................................................................................................................................................................................................................uccuaccccucccCuaccauuc..................   1    1    JCK-2
...................................................................................................................................................................................................................uccuacccAucccauaccauuc..................   1    1    JCK-3
...................................................................................................................................................................................................................uccuaccccucccauaccauuU..................   2    1    LT-3
...................................................................................................................................................................................................................uccuaccccucccauCccauuc..................   1    1    JCK-1
...................................................................................................................................................................................................................uccuaccccucccauaccauuU..................   3    1    JCK-1
...................................................................................................................................................................................................................uccuaccccucccauaccauuA..................   1    1    JCK-3
...................................................................................................................................................................................................................uccuaccccucccauaccauuU..................   5    1    JT-3
...................................................................................................................................................................................................................uccuCccccucccauaccauuc..................   1    1    JCK-2
...................................................................................................................................................................................................................uccuaccccucccauaccauuU..................   3    1    JCK-3
...................................................................................................................................................................................................................uccuaccccucccauaccauuc..................   23   0    LT-3
...................................................................................................................................................................................................................uccuaccccucccauaccauuc..................   66   0    JCK-1
...................................................................................................................................................................................................................uccuaccccuAccauaccauuc..................   1    1    JCK-1
....................................................................................................................................................................................................................ccuaccccucccauacca.....................   2    0    LT-2
....................................................................................................................................................................................................................ccuaccccucccauacca.....................   1    0    LT-3
....................................................................................................................................................................................................................ccuaccccucccauaccau....................   3    0    JCK-3
....................................................................................................................................................................................................................cUuaccccucccauaccauu...................   1    1    JCK-2
....................................................................................................................................................................................................................ccuaccccucccauaccauu...................   4    0    LT-2
....................................................................................................................................................................................................................ccuaccccucccauaccauu...................   1    0    LCK-3
....................................................................................................................................................................................................................ccuaccccucccauaccauu...................   20   0    JCK-2
....................................................................................................................................................................................................................ccuaccccucccauaccauu...................   3    0    LT-3
....................................................................................................................................................................................................................ccuaccccucccauaccauu...................   5    0    JCK-1
....................................................................................................................................................................................................................ccuaccccucccauaccauu...................   12   0    JCK-3
....................................................................................................................................................................................................................ccuaccccucccauaccauu...................   3    0    LT-1
....................................................................................................................................................................................................................ccuaccccucccauaccauuc..................   34   0    JCK-2
....................................................................................................................................................................................................................ccuaAcccucccauaccauuc..................   1    1    JCK-1
....................................................................................................................................................................................................................ccuaccccucccauaccauuU..................   5    1    JCK-3
....................................................................................................................................................................................................................ccuaccccucccauaccauuU..................   1    1    LT-3
....................................................................................................................................................................................................................ccuaccccucccauaccauuc..................   32   0    JCK-3
....................................................................................................................................................................................................................ccuaccccucccauaccauuU..................   5    1    JCK-2
....................................................................................................................................................................................................................ccuaccccCcccauaccauuc..................   1    1    JCK-2
....................................................................................................................................................................................................................ccuaccccucccauaccauuc..................   5    0    LT-3
....................................................................................................................................................................................................................ccuaccccucccauaccauuc..................   4    0    LT-1
....................................................................................................................................................................................................................ccuaccccucccauaccauuc..................   22   0    JCK-1
....................................................................................................................................................................................................................ccuaccccucccauaccauuU..................   2    1    JCK-1
....................................................................................................................................................................................................................ccuaccccucccauaccauucU.................   3    1    LT-2
....................................................................................................................................................................................................................ccuaccccucccauaccauuca.................   1    0    JCK-1
.....................................................................................................................................................................................................................cuaccccucccauaccau....................   3    0    LT-1
.....................................................................................................................................................................................................................cuaccccucccauaccauu...................   3    0    JCK-2
.....................................................................................................................................................................................................................cuaccccucccauaccauu...................   5    0    LT-3
.....................................................................................................................................................................................................................cuaccccucccauaccauu...................   4    0    LCK-3
.....................................................................................................................................................................................................................cuaccccucccauaccauu...................   6    0    LT-1
.....................................................................................................................................................................................................................cuaccccucccauaccauu...................   1    0    LT-2
.....................................................................................................................................................................................................................Uuaccccucccauaccauu...................   1    1    LT-1
.....................................................................................................................................................................................................................cuaccccucccauaccaCu...................   1    1    LT-2
.....................................................................................................................................................................................................................cuaccccucccauaccauuU..................   2    1    JCK-3
.....................................................................................................................................................................................................................cuaccccucccauaccauuc..................   6    0    LT-3
.....................................................................................................................................................................................................................cuaccccucccauaccauuc..................   3    0    JCK-1
.....................................................................................................................................................................................................................cuaccccucccauaccauuc..................   4    0    LT-1
.....................................................................................................................................................................................................................cuaccccucccauaccauuc..................   3    0    JCK-3
.....................................................................................................................................................................................................................cuaccccucccauaccauuc..................   5    0    LT-2
.....................................................................................................................................................................................................................cuCccccucccauaccauuc..................   1    1    LT-3
.....................................................................................................................................................................................................................cuaccccucccauaccauuc..................   5    0    JCK-2
.....................................................................................................................................................................................................................cuaccccucccauaccauuU..................   3    1    LCK-3
.....................................................................................................................................................................................................................cuaccccucccauaccauuU..................   1    1    LT-2
```

```
Star Mature
uuccuuuuuuucaccaaaaucuagacucaauuaauuucucuuuucccuuccuccaccaucugguguauuucuuuucucacuaaaaggauggaagagggaaaggcuuaagaguguggguggaagugauagguaugggggguuaggacagagagaaauacucucuuuuccccaauuuauuuuuuuuaauuggggggaagggaaugaauucuuuccuaccccucccauaccauucauuuccauccuuuccuuc
....................................................................................................................................................................................................................cuaNcccucccauaccauuc..................  1  1  JCK-1
....................................................................................................................................................................................................................cuaccccucccauaccauuU..................  1  1  JCK-2
....................................................................................................................................................................................................................cuaccccucccauaccauuU..................  1  1  LT-1
....................................................................................................................................................................................................................cuaccccucccauaccauuca.................  2  0  JCK-1
....................................................................................................................................................................................................................cuaccccucccauaccauucU.................  1  1  LCK-3
....................................................................................................................................................................................................................cuaccccucccauaccauuca.................  1  0  LT-1
.....................................................................................................................................................................................................................uaccccucccauaccauuc..................  1  0  LT-1
.............................................................................................................................................................................................................................ccauaccauucauuuccauccu.......  2  0  LT-1
.............................................................................................................................................................................................................................ccauaccauucauuuccauccuAu.....  1  1  JCK-2
.............................................................................................................................................................................................................................ccauaccauucauuuccauccuAu.....  1  1  JCK-3
..............................................................................................................................................................................................................................cauaccauucauuuccauc.........  1  0  LT-1
..............................................................................................................................................................................................................................cauaccauucauuuccauU.........  1  1  JCK-2
..............................................................................................................................................................................................................................cauaccauucauuuccauccu.......  1  0  LT-3
..............................................................................................................................................................................................................................cauaccauucauuuccauccuu......  2  0  JCK-3
..............................................................................................................................................................................................................................cauaccauucauuuccauccuu......  1  0  JCK-2
..............................................................................................................................................................................................................................cauaccauucauuuccauccuuu.....  1  0  JCK-2
..............................................................................................................................................................................................................................cauaccauucauuuccauccuuu.....  2  0  JCK-3
..............................................................................................................................................................................................................................cauaccauucauuuccauccuuuc....  2  0  JCK-1
...............................................................................................................................................................................................................................auaccauucauuuccauc.........  1  0  LT-3
...............................................................................................................................................................................................................................auaccauucauuuccauccuu......  1  0  LT-3
...............................................................................................................................................................................................................................auaccauucauuuccauccuuu.....  1  0  LT-3
...............................................................................................................................................................................................................................auaccauucauuuccauccuuuc....  1  0  LT-1
...............................................................................................................................................................................................................................auaccauucauuuccauccuuuc....  1  0  LT-2
...............................................................................................................................................................................................................................auaccauucauuuccauccuuuU....  1  1  JCK-3
...............................................................................................................................................................................................................................auaccauucauuuccauccuuucc...  3  0  JCK-2
...............................................................................................................................................................................................................................auaccauucauuuccCuccuuucc...  1  1  JCK-2
...............................................................................................................................................................................................................................auaccauucauuuccauccuuuccuu.  1  0  LT-3
................................................................................................................................................................................................................................uaccauucauuuccauccuuucc...  2  0  JCK-1
....................................................................................................................................................................................................................................auucauuuccauccuuucc...  2  0  LT-3
....................................................................................................................................................................................................................................auucauuuccauccuuuccu..  2  0  LT-2
....................................................................................................................................................................................................................................auucauuuccauccuuuccuu.  3  0  LT-3
```
